# Prevalence and trends of *Clostridioides difficile* infection among persons requiring maintenance hemodialysis: A systematic review and meta-analysis

**DOI:** 10.1017/ice.2022.217

**Published:** 2023-07

**Authors:** Yousef M. Elfanagely, Joshua Ray Tanzer, Anuoluwapo Shobayo, Mouhand F.H. Mohamed, Jonathan J.C. Ho, Douglas Shemin, Laura Pavlech, Erika M.C. D’Agata

**Affiliations:** 1 Department of Internal Medicine, Warren Alpert School of Medicine, Brown University, Providence, Rhode Island; 2 Department of Biostatistics, Warren Alpert School of Medicine, Brown University, Providence, Rhode Island; 3 Division of Infectious Diseases, Warren Alpert School of Medicine, Brown University, Providence, Rhode Island; 4 Division of Nephrology, Warren Alpert School of Medicine, Brown University, Providence, Rhode Island; 5 Brown University Library, Providence, Rhode Island

## Abstract

**Objective:**

*Clostridioides difficile* infection (CDI) is among the most common cause of healthcare-associated infections. Persons requiring maintenance hemodialysis (MHD) are at increased risk of CDI and associated mortality compared to persons not requiring MHD. Given the clinical impact of CDI among persons requiring MHD, we aimed to quantify the burden of CDI and trends over time in this patient population.

**Study design:**

A systematic review and meta-analysis of studies reporting rates of CDI among persons requiring MHD in MEDLINE, Embase, Web of Science Core Collection, CINAHL, and Cochrane Central Register of Controlled Trials were performed. Searches were conducted on May 17, 2021, and March 4, 2022.

**Results:**

In total, 2,408 titles and abstracts were identified; 240 underwent full text review. Among them, 15 studies provided data on rates of CDI among persons requiring MHD, and 8 of these also provided rates among persons not requiring MHD. The pooled prevalence of CDI among persons requiring MHD was 19.14%, compared to 5.16% among persons not requiring MHD (odds ratio [OR], 4.35; 95% confidence interval [CI], 2.07–9.16; *P* = .47). The linear increase in CDI over time was significant, increasing an average of 31.97% annually between 1993 and 2017 (OR, 1.32; 95% CI, 1.1–1.58; *P* < .01). The linear annual increase was similar among persons requiring and not requiring MHD (OR, 1.28; 95% CI, 1.13–1.45; *P* = .11).

**Conclusions:**

Persons requiring MHD have a 4-fold higher risk of CDI compared to persons not requiring MHD, and rates of CDI are increasing over time in both groups.

*Clostridioides difficile* infections (CDIs) are associated with considerable morbidity and mortality. In 2017, there were almost half a million CDIs and >20,000 associated deaths in the United States.^1–3^ CDI is the most common cause of gastroenteritis-related deaths and is among the most common cause of healthcare-associated infections.^3,4^ Rates of CDI have been increasing both in the hospital and community settings.^5^

Persons with chronic kidney disease and those on maintenance hemodialysis (MHD) are at high-risk of CDI due to substantial antimicrobial exposure, frequent hospitalizations, and immune dysfunction.^6^ Rates of CDI and associated mortality are up to 2-fold higher among persons with chronic kidney disease compared to the general population, and the requirement for MHD increases these rates even further.^7–10^

Given the clinical significance of CDI among persons requiring MHD and increasing CDI rates in healthcare settings, we evaluated the burden of CDI, potential risk factors for CDI, mortality rates, and mortality-associated risk factors among persons requiring MHD. We performed a systematic review of the literature that reported CDI prevalence among persons requiring MHD and a meta-analysis to quantify it. We compared the prevalence of CDI among persons requiring MHD with that of persons not requiring MHD. Trends of CDI over time among both groups were also evaluated.

## Materials and methods

This systematic review and meta-analysis were performed according to the PRISMA guidelines.^11^ The study did not require institutional review board nor ethics committee approval because all data were publicly available.

### Search strategy

A medical librarian constructed comprehensive search strategies for each of the following databases: MEDLINE, Embase, Web of Science Core Collection, CINAHL, and Cochrane Central Register of Controlled Trials. The search strategies used a combination of controlled vocabulary terms and keywords to describe 2 concepts: *Clostridioides difficile* and hemodialysis or chronic or end stage kidney disease (Supplementary Material online). All databases were searched on May 17, 2021, and March 4, 2022, with the exception of Web of Science Core Collection, which was only searched on May 17, 2021, because access to this database was no longer available on March 4, 2022. The reference lists of and citations to key articles were reviewed to identify additional studies.

Results were exported to EndNote for Windows version X9.3.3 software (Clarivate Analytics, Philadelphia, PA), and duplicates were removed using a previously described method.^12^ The deduplicated results were uploaded to Covidence (Veritas Health Innovations, Melbourne, Australia) for screening.

### Selection criteria

*Inclusion criteria.* The following inclusion criteria were applied:

1. Studies published in English
2. Studies with a patient population that included patients with stage 5 chronic kidney disease, with end-stage renal disease, or on maintenance hemodialysis
3. Studies that reported symptomatic *Clostridioides difficile* infection as an outcome
4. Studies providing data on CDI rates among all persons on MHD
5. Randomized or nonrandomized controlled trials, cohort studies, and case–control studies.

*Exclusion criteria.* We applied the following exclusion criteria:

1. Meta-analyses, letters, case reports, commentaries, conference abstracts, and CDI outbreaks
2. Studies with duplicate results or those that continued work from previous publications
3. Studies with pediatric populations, peritoneal dialysis, or nursing home populations
4. Studies that addressed *C. difficile* colonization or used a presumptive diagnosis of CDI based on clinical suspicion or diarrhea, and not laboratory confirmation
5. Studies in which differentiation between acute hemodialysis, chronic kidney disease, and maintenance hemodialysis could not be confirmed
6. Studies that reported rates among persons requiring and not requiring MHD among persons with CDI, in contrast to rates of CDI among persons requiring and not requiring MHD.

### Data extraction

Four researchers (Y.E., A.S., M.M., and J.H.) independently screened the literature and cross-checked the articles. Senior researchers (E.M.C.D. and J.R.T.) resolved discrepancies. After selecting which studies would be included, data were extracted, including first author’s name, year of publication, study population and location, study design, and number of persons requiring and not requiring MHD with and without CDI.

### Quality assessment

Two reviewers (Y.E. and A.S.) appraised the quality of the studies, and these appraisals were confirmed by a third reviewer (E.M.C.D.). The Newcastle-Ottawa Scale, a star-based rating system of 9 domains, was used to assess the methodological quality of the included studies.^13^ The 2 comparability fields were not relevant to this analysis and were not included; thus, each study could receive a maximum of 7 stars. To consider the possibility of publication bias, funnel plots were visually examined and the Egger test was performed.^14^

### Data synthesis and statistical analyses

*Effect sizes.* For the primary research question, we compared the risks of CDI between persons on MHD and persons not requiring MHD. This focused, 2-group comparison was selected so that persons not requiring MHD within the study samples could act as a general control. According to the study inclusion criteria, all studies reported rates of CDI among persons on MHD; however, some studies exclusively described persons on MHD. For this reason, the effect size of interest was the log odds risk of infection, defined as 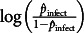 where 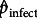 is the estimated probability of CDI. This effect size was selected because estimated risks could be directly compared between persons on MHD and controls without having to combine estimates into a single metric (eg, risk ratio or risk difference), which would induce a missing-data problem based on how sampling was performed. Additionally, the log odds can be better approximated as normally distributed, making it more conductive to statistical modeling.^15^

In each study, the log odds of infection was estimated from the reported summary data for persons on MHD and all other patients if data were available. Variances of estimates were approximated using the Monte Carlo simulation.^16^ For each log odds estimate, 1,000 random samples of the same size and estimated probability 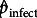 were generated from a binomial distribution and were transformed into the log odds. The variance among the simulated log odds values was used as the variance of the estimate for the final meta-analysis model.

*Analysis plan.* A random-effects model was used to pool the effect sizes. This model was selected because many of the studies were conducted in different locations with different sample characteristics, so the assumed homogeneity of a fixed-effects model did not seem appropriate. Additionally, because many of the studies provided both estimates of risk of CDI for both persons on MHD and persons not requiring MHD, random-effects modeling is well equipped analytically to address heterogeneity of variances and correlations between different observations nested within studies.^17^

The fixed effects in the model included year as a continuous value and MHD status as a binary indicator. For studies that were conducted for ≥1 year, the midpoint was used. We hypothesized that persons on MHD would be at greater risk of CDI and that risks of CDI would increase year after year. Trajectories over time between persons on MHD and controls were specifically tested within the model.

## Results

### Study identification and selection

**Fig. 1.**
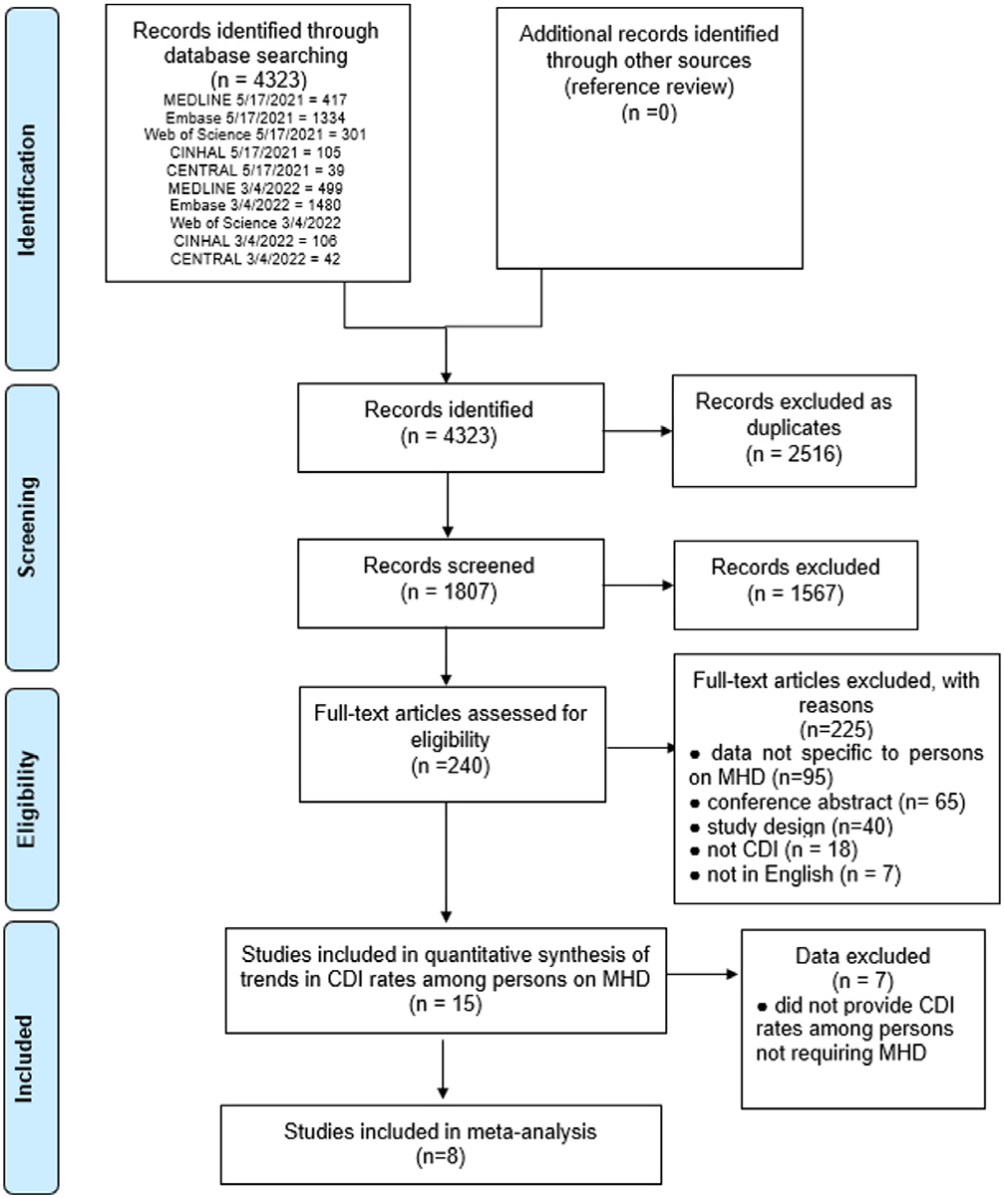
Preferred Reporting Items for Systematic Reviews and Meta-Analyses (PRISMA) flow chart of the study search and review process examining CDI among persons on maintenance hemodialysis. Note. CDI, *Clostridioides difficile* infection; MHD, maintenance hemodialysis.

The details of the selection process are summarized in the flow diagram (Fig. 1). The initial electronic search yielded 2,208 studies. After the removal of duplicates, 1,811 studies were screened by title and abstract. In total, 1,571 studies were excluded, and 240 studies were assessed based on full texts. Subsequently, 225 studies were excluded, a large proportion of which were excluded due to the inability to determine whether hemodialysis referred to acute or maintenance dialysis and/or to the number of persons on MHD among those with chronic kidney disease. Also, 15 studies were included in the quantitative analysis of trends in CDI rates over time among persons on MHD.^1,7,8,18–29^ Furthermore, 8 of these studies also provided CDI prevalence data among persons not requiring MHD and were included in the meta-analysis.^7,18,19,22,23,25,27,29^

### Study characteristics

Characteristics of the 15 studies are summarized in Table 1, including country, study design, and patient population. Of the 15 studies included in the trend analysis of CDI rates, 13 were conducted among hospitalized patients.^7,8,18–27,29^ In 2 studies, the proportion of patients in whom CDI was diagnosed either in the outpatient or hospital setting could not be determined.^1,28^ All 8 studies included in the meta-analysis were conducted in a hospital setting.

**Table 1.** Characteristics of Studies Included in the Systematic Review and Meta-analysis

First Author and Year	Years of Data	Country	Study Design	Population	Included in Meta-analysis?	No. of Persons With CDI/Total Persons (%)	
						MHD	Non-MHD
D’Agata 2000	1995–97	USA	Retrospective case–control	Inpatient	Yes	3/5182 (0.06)	65/398,983 (0.02) Hospitalized persons
Demir 2018	2014–15	Canada	Retrospective cohort	Inpatient	Yes	6/10 (60.0)	27/238 (11.3) Hospitalized persons
Do 1998	1993–94	Canada	Retrospective case–control	Inpatient	No	2/59 (3.4)	N/A
Eddi 2010	2006–07	USA	Retrospective case–control	Inpatient	No	17/31 (54.8)	N/A)
Eui Oh 2013	2008–09	Korea	Retrospective case–control	Inpatient	Yes	16/37 (43.2)	69/366 (18.8) Hospitalized persons with diarrhea
Guh 2017	2014	USA	Prospective case–control	Outpatient and inpatient	No	5/5 (100)	N/A
Huang 2014	2008–09	China	Retrospective case–control	Inpatient	Yes	9/32 (28.2)	81/7782 (1.0) Hospitalized persons
Keddis 2012	2005–09	USA	Retrospective cohort	Inpatient (National Hospital Discharge Survey)	Yes	1,281,132/ 2,945,130 (43.5)	1,418,723/5,079,634 (27.9) Hospitalized persons with CKD
Kim 2016	2010–13	Korea	Retrospective case–control	Inpatient	No	22/32 (68.8)	N/A
Morfin 2018	2014–16	Mexico	Retrospective case–control	Inpatient	Yes	82/232 (35.3)	272/1006 (27.0) Hospitalized persons with CKD and diarrhea
Pant 2012	2009	USA	Retrospective case–control	Inpatient (Nationwide database)	No	5,151/184,139 (2.8)	N/A
Predrag 2016	2013–14	Serbia	Prospective case–control	Inpatient	Yes	7/9 (77.8)	30/102 (29.4) Hospitalized persons with diarrhea
Sheth 2010	1999–2017	USA	Retrospective case–control	Outpatient and inpatient	No	28/196 (14.3)	N/A
Tirath 2017	2005–08	USA	Retrospective case–control	Inpatient (US Renal Data System)	No	17,853/ 419,875 (4.3)	N/A
Wei 2015	2009	Taiwan	Retrospective case–control	Inpatient	Yes	2/9 (22.2)	4/140 (0.01) Hospitalized persons

Note. CDI, *Clostridioides difficile* infection; MHD, maintenance hemodialysis; CKD, chronic kidney disease; N/A, not available.

### Risk of bias and quality assessment

The reviewers were in complete agreement that all studies were suitable for use in the systematic review. All studies included in the meta-analysis were deemed of high quality, with Newcastle Ottawa Scale scores of 7 of 7 stars for 8 studies and 6 of 7 stars for 2 studies (Supplementary Tables 1 and 2 online).

To assess publication bias, heterogeneity, and chance, a funnel plot was constructed to compare the risks of CDI to the precision of estimate (Supplementary Fig. 1 online). Most participants not on MHD demonstrated minimal change in risk regardless of precision; however, the risks also tended to be larger, making them more robust to chance findings. Among persons on MHD, we detected a visual trend such that more extreme risks tended to be observed in less precise samples. The Egger test did not indicate bias within controls (Z = 0.57; *P* = .5700); however, the Egger results were much closer to significance among persons on MHD (Z = 1.58; *P* = .1137). This trend was likely attributable to the high variation in estimate precision and modest sample size. After model estimation was complete, the more extreme observations and studies with disproportionately large sample sizes were removed from the analysis to assess the influence of these chance observations. This removal did not result in any changes to inference (data not shown).

### Systematic review of CDI rates, risk factors and outcomes

Table 1 provides the percentage of CDI among persons requiring MHD and not requiring MHD. Among persons requiring MHD, CDI ranged from 0.05% to 77.8%. In a matched case–control study of 452 subjects, all 5 persons requiring MHD developed CDI.^1^ Among the 10 studies that compared CDI rates among persons on MHD to those not requiring MHD, 9 identified requirement for MHD as an independent risk factor for CDI, with adjusted odds ratios ranging from 1.33 (95% confidence interval [CI], 1.32–1.35) to 13.5 (95% CI, 2.85–63.8).^7,19,21–25,27,29^

Two studies analyzed risk factors for CDI among persons requiring MHD.^8,28^ In 2010, Sheth et al^28^ identified a serum albumin ≤3 g/dL (adjusted hazard ratio [aHR], 3.03; 95% CI, 1.75–5.55) and a higher Charlson comorbidity index (aHR, 1.17; 95% CI, 1.00–1.36) as significantly associated with a higher risk of CDI.^28^ In 2017, Tirath et al^8^ identified 3 comorbidities associated with the greatest risk for CDI: age ≥65 years (adjusted risk ratio [aRR], 1.76; 95% CI, 1.7–1.82), human immunodeficiency virus (aRR, 2.68; 95% CI, 2.4–2.99), and bacteremia (aRR, 1.74; 95% CI, 1.68–1.80).^8^ In this study, Hispanic ethnicity (aRR, 0.76; 95% CI, 0.72–0.70) and Black race (aRR, 0.75; 95% CI, 0.73–0.78) were associated with a decreased risk of CDI.^8^

Three studies compared mortality among persons on MHD with and without CDI, which ranged from 13.2% to 68.8%.^8,26,28^ In 2 studies, mortality was significantly higher among persons requiring MHD with a 2-fold higher risk of death.^8,24^ In the study by Tirath et al,^8^ independent factors associated with mortality included age ≥65 years (aHR, 2.28) and presence of cirrhosis (aHR, 1.76); however, confidence intervals were not provided.^8^

In 2012, Pant et al^26^ reported other outcomes associated with CDI among persons requiring MHD. Length of hospital stay was greater among persons on MHD with CDI (mean difference, 9.4 days; 95% CI, 9.2–9.5) and hospital costs were higher (mean difference, $62,824; 95% CI, 61,615–64,033).^26^

In contrast to the studies that compared rates among persons not requiring MHD, Keddis et al^7^ compared mortality rates and other outcomes associated with CDI between persons requiring MHD and persons with chronic kidney disease (CKD) not requiring MHD. They detected no differences in length of hospital stays between the 2 groups, and lower rates of colectomy were detected among persons with CDI requiring MHD (adjusted odds ratio [aOR], 0.327; 95% CI, 0.26–0.40).

### Meta-analysis and trends in CDI over time

**Fig. 2.**
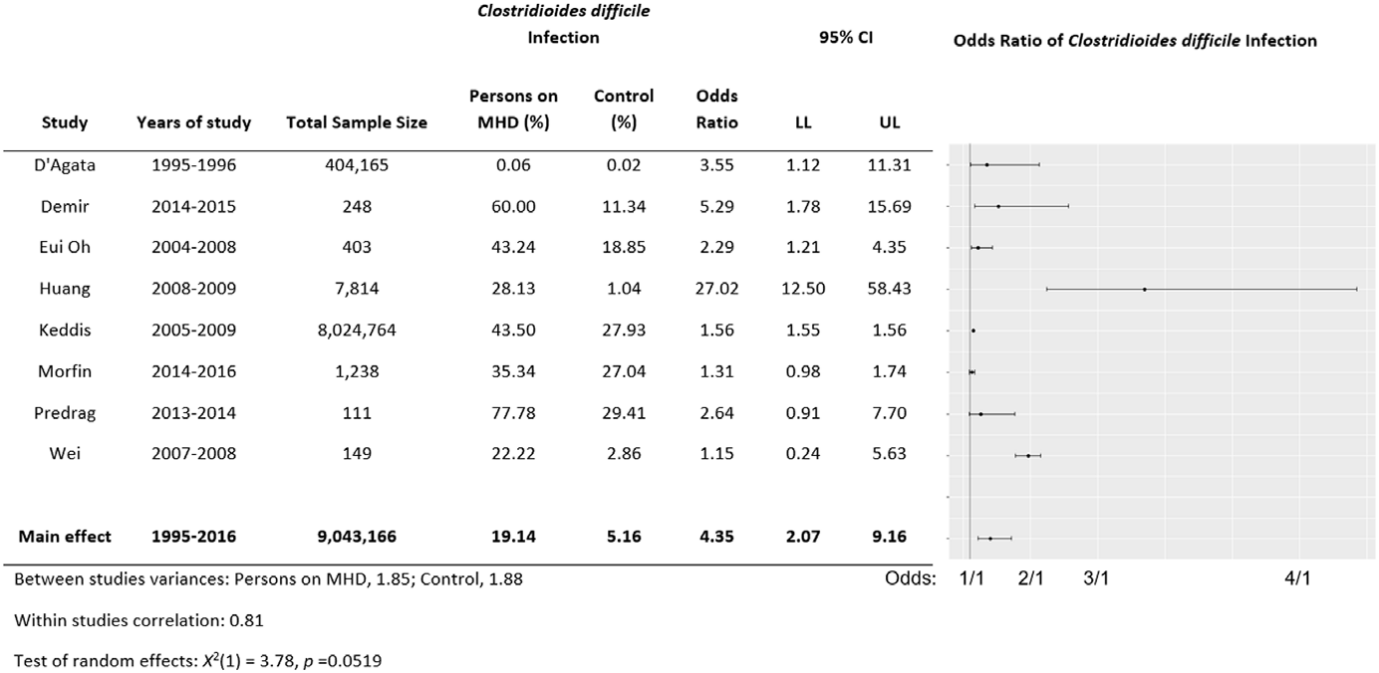
Forest plot of the 10 included studies providing *Clostridioides difficile* prevalence among persons requiring and not requiring maintenance hemodialysis.

Figure 2 shows the forest plot reporting individual study prevalence and odds ratios among the 8 studies providing estimates for both persons requiring and not requiring MHD.^7,18,19,22,23,25,27,29^ The pooled prevalence of CDI among persons requiring MHD was 19.14%, and for persons not requiring MHD, the pooled prevalence of CDI was 5.16%. Overall, persons requiring MHD had a 4-fold increased risk of CDI compared to persons not requiring MHD (OR, 4.35; 95% CI, 2.07–9.16; *P* = .47). The exclusion of the study by Keddis et al,^7^ which included a large patient population that may have influenced the study results, had a minimal impact on the results of the meta-analysis (data not shown).

**Fig. 3.**
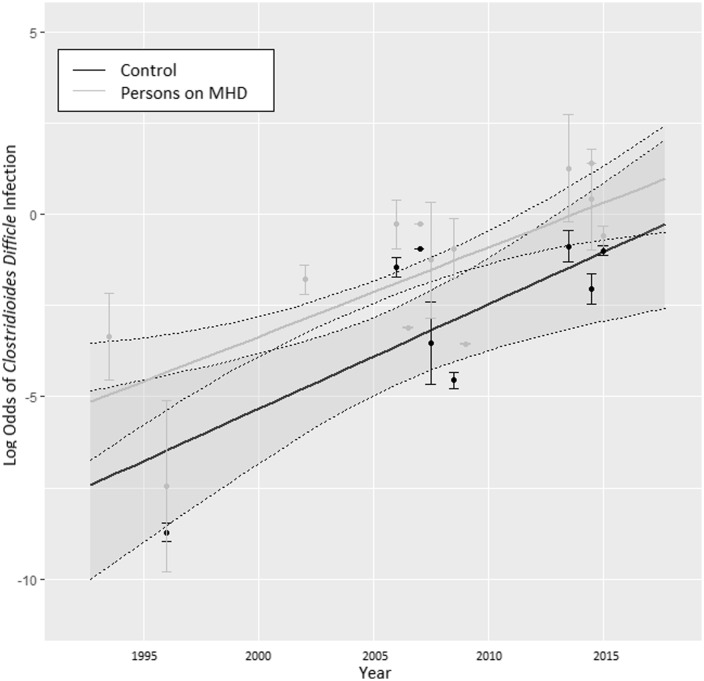
Trends in *Clostridioides difficile* infection over time comparing persons requiring and not requiring maintenance hemodialysis. The line and shaded regions represent the modeled average and 95% confidence intervals.

The trend analysis included 15 studies providing data on CDI prevalence among persons requiring MHD from 1993 to 2017^1,7,8,18–29^ and 8 studies^7,18,19,22,23,25,27,29^ providing data on CDI prevalence among persons not requiring MHD from 1995 to 2016 (Fig. 3). For both groups, the linear increase in CDI risk over time was significant, increasing 31.97% on average annually during the study period (OR, 1.32; 95% CI, 1.1–1.58; *P* < .01). The linear annual increase in risk among persons requiring MHD was similar to that of persons not requiring MHD (OR, 1.28; 95% CI, 1.13–1.45; *P* = 0.11).

## Discussion

We performed a systematic review and meta-analysis to determine the rates and trends over time of CDI among persons requiring MHD compared to persons not requiring MHD and to provide a qualitative analysis of risk factors and mortality associated with CDI among persons on MHD.

Rates of CDI were 4-fold higher among persons requiring MHD compared to persons not requiring MHD, with pooled prevalences of 19.14% and 5.16%, respectively. The trend analysis, from 1993 to 2017, demonstrated that there was a significant increase in rates of CDI over time in both the groups of persons who did and did not require MHD, increasing by an average of 31.97%, annually. The rise in trajectories was parallel between the 2 groups. Notably, CDI rates among persons not requiring MHD were very high in some studies, but this finding reflects the fact that studies with this population included persons with diarrhea or chronic kidney disease who were at higher risk of CDI.

Antimicrobial exposure and hospitalizations are among the main risk factors for an increased risk of CDI among all patients.^2^ In this systematic review, risk factors specific to the MHD population included age ≥65 years, serum albumin ≤3 g/dL, higher Charlson comorbidity index, human immunodeficiency virus (HIV), and bloodstream infections.^8,28^ Mortality associated with CDI was 2-fold higher among persons requiring MHD compared to persons not requiring MHD, and ranged from 13.2% to 68.8%.^8,26,28^ Risk factors for CDI-associated mortality included age ≥65 years and presence of cirrhosis.^8^ Lastly, a diagnosis of CDI increased the length of hospital stay by 9 days, with increased hospital costs among persons requiring MHD.^26^

The substantially higher and rising rates of CDI among persons requiring MHD and associated higher morbidity and mortality compared to persons not requiring MHD both emphasize the importance of preventing *C. difficile* spread and CDI in this patient population.

The Nephrologists Transforming Dialysis Safety work group, an initiative funded by the Centers for Disease Control and Prevention in collaboration with the American Society of Nephrology, recently published recommendations for the prevention of *C. difficile* spread in outpatient dialysis facilities.^30^ The strategies outlined parallel those for multidrug-resistant organisms; the transmission dynamics of *C. difficile* are very similar. One major difference, however, is that *C. difficile* produces spores that survive on inanimate surfaces for many months.^31,32^ These spores are resistant to a variety of routinely used environmental disinfectants and require specific *C. difficile* sporicidal agents registered by the Environmental Protective Agency.^33^ Hand hygiene measures also differ. Soap and water substantially reduces *C. difficile* spores, in contrast to alcohol-based sanitizers.^31,32,34^ Thus, soap and water is the preferred hand hygiene measure to prevent *C. difficile* spread in outpatient dialysis units.^30^ However, given the greater compliance with alcohol-based sanitizers, this hand hygiene measure is an alternative, except when there is visible hand soiling, during CDI outbreaks, or when there is concern for spread of CDI within the dialysis facility.^30^

Compliance with the Nephrologists Transforming Dialysis Safety strategies aimed at preventing the spread of *C. difficile* should be monitored in outpatient dialysis facilities to prevent de novo acquisition and *C. difficile* outbreaks. Several outbreaks in outpatient dialysis facilities have been reported; they have affected a substantial number of patients within the dialysis unit in addition to dialysis healthcare workers.^35,36^ Implementation of *C. difficile*–specific infection prevention strategies has led to the end of CDI outbreaks.^35^

Many of the risk factors for CDI are modifiable, especially antimicrobial exposure. Up to 30% of antimicrobial doses administered in the outpatient dialysis facility are not indicated according to national guidelines.^37^ Implementation of antimicrobial stewardship programs in dialysis facilities has been shown to significantly reduce antimicrobial prescribing. These programs have yielded substantial decreases in infections and mortality caused by *C. difficile* and multidrug-resistant organisms without negative outcomes such as increased hospitalizations or bloodstream infections.^38,39^

This review had several limitations. First, most studies were based in the hospital setting and did not distinguish between acquisition of CDI during a hospitalization versus the presence of CDI at hospital admission. Thus, we were unable to quantify the prevalence of CDI in the hospital versus the dialysis unit setting. Second, hospitals or outpatient dialysis settings with high CDI prevalence could be more likely to publish studies of CDI rates, which would introduce a selection bias toward higher rates. Third, only 3 studies meeting the inclusion and exclusion criteria reported rates of mortality associated with CDI among persons requiring MHD; therefore, a meta-analysis could not be performed. Lastly, changes in diagnostic testing for CDI over the study period, such as nucleic acid amplification tests, may have identified colonization in the presence of diarrhea from other causes, leading to higher reported rates of CDI in studies published after 2005.

In summary, *C. difficile* causes a substantial burden on persons requiring MHD, with higher mortality rates compared to persons not requiring MHD. Adherence to *C. difficile*–specific infection prevention recommendations and improving antimicrobial prescribing patterns are some of the important strategies to limit *C. difficile* spread in the population of patients requiring MHD.

## Supplementary material

For supplementary material accompanying this paper visit https://doi.org/10.1017/ice.2022.217. click here to view supplementary material
