# Supplementary material for: Prevalence and trends of Clostridioides difficile infection among persons requiring maintenance hemodialysis: A systematic review and meta-analysis
Source: Infect Control Hosp Epidemiol. 2022 Sep 23;44(7):1068–75. doi: 10.1017/ice.2022.217 (PMC10369223; doi:10.1017/ice.2022.217)
Supplement: Supplementary file 1 [file S0899823X22002173sup.zip › S0899823X22002173sup001.docx]

**SUPPLEMENTARY MATERIAL**

**Search Strategies for Bibliographic Databases**

**MEDLINE Search Strategy**

**Platform**: Ovid

**Years Searched**: Ovid MEDLINE and Epub Ahead of Print, In-Process & Other Non-Indexed Citations and Daily 1946 to May 14, 2021 and March 3, 2022

**Limits**: Humans (search hedge), English language

**Dates Searched:** May 17, 2021; March 4, 2022

**Number of Results:** 417; 499

| 1 | exp clostridioides/ |
| --- | --- |
| 2 | clostridium/ |
| 3 | exp clostridium infections/ |
| 4 | ((bacillus or clostrid$ or peptoclostrid$ or pseudomembran$) adj2 (colitis or dif?icile or dif?icilis or diarrh?ea or disease$ or enteritis or enterocolitis or infect$)).ti,ab,kw. |
| 5 | clostridioses.ti,ab,kw. |
| 6 | clostridiosis.ti,ab,kw. |
| 7 | c diff.ti,ab,kw. |
| 8 | CDAD.ti,ab,kw. |
| 9 | or/1-8 |
| 10 | exp renal replacement therapy/ |
| 11 | ultrafiltration/ |
| 12 | (renal replac$ adj2 (continuous or therap$)).ti,ab,kw. |
| 13 | dialys$.ti,ab,kw. |
| 14 | h?emodiafiltrat$.ti,ab,kw. |
| 15 | h?emodialys$.ti,ab,kw. |
| 16 | h?emofiltrat$.ti,ab,kw. |
| 17 | (acetate free adj2 biofiltrat$).ti,ab,kw. |
| 18 | ultrafiltrat$.ti,ab,kw. |
| 19 | CAVHD.ti,ab,kw. |
| 20 | CRRT.ti,ab,kw. |
| 21 | CVVH.ti,ab,kw. |
| 22 | CVVHD.ti,ab,kw. |
| 23 | CVVHDF.ti,ab,kw. |
| 24 | SCUF.ti,ab,kw. |
| 25 | exp renal insufficiency, chronic/ |
| 26 | ((kidney or renal) adj2 (chronic or end stage or endstage)).ti,ab,kw. |
| 27 | CKD.ti,ab,kw. |
| 28 | CKF.ti,ab,kw. |
| 29 | CRD.ti,ab,kw. |
| 30 | CRF.ti,ab,kw. |
| 31 | ESKD.ti,ab,kw. |
| 32 | ESKF.ti,ab,kw. |
| 33 | ESRD.ti,ab,kw. |
| 34 | ESRF.ti,ab,kw. |
| 35 | or/10-34 |
| 36 | and/9,35 |
| 37 | exp animals/ not humans/ |
| 38 | 36 not 37 |
| 39 | limit 38 to english language |

**Embase Search Strategy**

**Platform**: Elsevier

**Years Searched**: 1947 – Present

**Limits**: Humans (search hedge), English language

**Dates Searched:** May 17, 2021; March 4, 2022

**Number of Results:** 1334; 1480

| 1 | 'clostridioides'/exp |
| --- | --- |
| 2 | 'clostridium'/de |
| 3 | 'clostridium infection'/exp |
| 4 | 'pseudomembranous colitis'/de |
| 5 | ((bacillus OR clostrid* OR peptoclostrid* OR pseudomembran*) NEAR/2 (colitis OR difficile OR dificile OR difficilis OR dificilis OR diarrhea OR diarrhoea OR disease* OR enteritis OR enterocolitis OR infect*)):ti,ab,kw |
| 6 | 'clostridioses':ti,ab,kw |
| 7 | 'clostridiosis':ti,ab,kw |
| 8 | 'c diff':ti,ab,kw |
| 9 | 'cdad':ti,ab,kw |
| 10 | #1 OR #2 OR #3 OR #4 OR #5 OR #6 OR #7 OR #8 OR #9 |
| 11 | 'renal replacement therapy'/exp |
| 12 | 'ultrafiltration'/de |
| 13 | ('renal replac*' NEAR/2 (continuous OR therap*)):ti,ab,kw |
| 14 | 'dialys*':ti,ab,kw |
| 15 | 'haemodiafiltrat*':ti,ab,kw |
| 16 | 'hemodiafiltrat*':ti,ab,kw |
| 17 | 'haemodialys*':ti,ab,kw |
| 18 | 'hemodialys*':ti,ab,kw |
| 19 | 'haemofiltrat*':ti,ab,kw |
| 20 | 'hemofiltrat*':ti,ab,kw |
| 21 | ('acetate free' NEAR/2 biofiltrat*):ti,ab,kw |
| 22 | 'ultrafiltrat*':ti,ab,kw |
| 23 | 'cavhd':ti,ab,kw |
| 24 | 'crrt':ti,ab,kw |
| 25 | 'cvvh':ti,ab,kw |
| 26 | 'cvvhd':ti,ab,kw |
| 27 | 'cvvhdf':ti,ab,kw |
| 28 | 'scuf':ti,ab,kw |
| 29 | 'chronic kidney failure'/exp |
| 30 | ((kidney OR renal) NEAR/2 (chronic OR 'end stage' OR endstage)):ti,ab,kw |
| 31 | 'ckd':ti,ab,kw |
| 32 | 'ckf':ti,ab,kw |
| 33 | 'crd':ti,ab,kw |
| 34 | 'crf':ti,ab,kw |
| 35 | 'eskd':ti,ab,kw |
| 36 | 'eskf':ti,ab,kw |
| 37 | 'esrd':ti,ab,kw |
| 38 | 'esrf':ti,ab,kw |
| 39 | #11 OR #12 OR #13 OR #14 OR #15 OR #16 OR #17 OR #18 OR #19 OR #20 OR #21 OR #22 OR #23 OR #24 OR #25 OR #26 OR #27 OR #28 OR #29 OR #30 OR #31 OR #32 OR #33 OR #34 OR #35 OR #36 OR #37 OR #38 |
| 40 | #10 AND #39 |
| 41 | [animals]/lim NOT [humans]/lim |
| 42 | #40 NOT #41 |
| 43 | #40 NOT #41 AND [english]/lim |

**Web of Science Core Collection Search Strategy**

**Platform**: Clarivate Analytics

**Years Searched**: Science Citation Index Expanded, 1900-Present; Social Sciences Citation Index, 1900-Present; Arts & Humanities Citation Index, 1975-Present; Emerging Sources Citation Index, 2015-Present

**Limits**: None

**Date Searched:** May 17, 2021; did not search on March 4, 2022 because no longer had access to this database

**Number of Results:** 301

| 1 | TS=((bacillus  OR  clostrid*  OR  peptoclostrid*  OR  pseudomembran*)  NEAR/2  (colitis OR difficile OR dificile OR difficilis OR dificilis OR diarrhea OR diarrhoea OR disease* OR enteritis OR enterocolitis OR infect*) ) |
| --- | --- |
| 2 | TS=(clostridioses) |
| 3 | TS=(clostridiosis) |
| 4 | TS=("c  diff") |
| 5 | TS=(cdad) |
| 6 | #5 OR #4 OR #3 OR #2 OR #1 |
| 7 | TS=("renal  replac*"  NEAR/2  (continuous OR therap*) ) |
| 8 | TS=(dialys*) |
| 9 | TS=(h$emodiafiltrat*) |
| 10 | TS=(h$emodialys*) |
| 11 | TS=(h$emofiltrat*) |
| 12 | TS=("acetate  free"  NEAR/2  biofiltrat*) |
| 13 | TS=(ultrafiltrat*) |
| 14 | TS=(cavhd) |
| 15 | TS=(crrt) |
| 16 | TS=(cvvh) |
| 17 | TS=(cvvhd) |
| 18 | TS=(cvvhdf) |
| 19 | TS=(scuf) |
| 20 | TS=((kidney  OR  renal)  NEAR/2  (chronic OR "end stage" OR endstage) ) |
| 21 | TS=(ckd) |
| 22 | TS=(ckf) |
| 23 | TS=(crd) |
| 24 | TS=(crf) |
| 25 | TS=(eskd) |
| 26 | TS=(eskf) |
| 27 | TS=(esrd) |
| 28 | TS=(esrf) |
| 29 | #28 OR #27 OR #26 OR #25 OR #24 OR #23 OR #22 OR #21 OR #20 OR #19 OR #18 OR #17 OR #16 OR #15 OR #14 OR #13 OR #12 OR #11 OR #10 OR #9 OR #8 OR #7 |
| 30 | #29 AND #6 |

**CINAHL Plus with Full Text Search Strategy**

**Platform**: EBSCO

**Years Searched**: 1937 – Present; 1981 – Present

**Limits**: English language

**Dates Searched:** May 17, 2021; March 4, 2022

**Number of Results:** 105; 106

| S1 | (MH "Clostridium+") |
| --- | --- |
| S2 | (MH "Clostridium Infections+") |
| S3 | ( (TI(bacillus OR clostrid* OR peptoclostrid* OR pseudomembran*) N2 (colitis OR dif#icile OR dif#icilis OR diarrh#ea OR disease* OR enteritis OR enterocolitis OR infect*)) ) OR ( (AB(bacillus OR clostrid* OR peptoclostrid* OR pseudomembran*) N2 (colitis OR dif#icile OR dif#icilis OR diarrh#ea OR disease* OR enteritis OR enterocolitis OR infect*)) ) |
| S4 | (TI "clostridioses") OR (AB "clostridioses") |
| S5 | (TI "clostridiosis") OR (AB "clostridiosis") |
| S6 | (TI "c diff") OR (AB "c diff") |
| S7 | (TI "cdad") OR (AB "cdad") |
| S8 | S1 OR S2 OR S3 OR S4 OR S5 OR S6 OR S7 |
| S9 | (MH "Renal Replacement Therapy+") |
| S10 | (MH "Ultrafiltration") |
| S11 | ( (TI("renal replac*" N2 (continuous OR therap*)) ) OR ( (AB("renal replac*" N2 (continuous OR therap*)) ) |
| S12 | (TI "dialys*") OR (AB "dialys*") |
| S13 | (TI "h#emodiafiltrat*") OR (AB "h#emodiafiltrat*") |
| S14 | (TI "h#emodialys*") OR (AB "h#emodialys*") |
| S15 | (TI "h#emofiltrat*") OR (AB "h#emofiltrat*") |
| S16 | (TI("acetate free" N2 biofiltrat*)) OR (AB("acetate free" N2 biofiltrat*)) |
| S17 | (TI "ultrafiltrat*") OR (AB "ultrafiltrat*") |
| S18 | (TI "cavhd") OR (AB "cavhd") |
| S19 | (TI "crrt") OR (AB "crrt") |
| S20 | (TI "cvvh") OR (AB "cvvh") |
| S21 | (TI "cvvhd") OR (AB "cvvhd") |
| S22 | (TI "cvvhdf") OR (AB "cvvhdf") |
| S23 | (TI "scuf") OR (AB "scuf") |
| S24 | (MH "Renal Insufficiency, Chronic+") |
| S25 | ( (TI (kidney OR renal) N2 (chronic OR "end stage" OR endstage)) ) OR ( (AB (kidney OR renal) N2 (chronic OR "end stage" OR endstage)) ) |
| S26 | (TI "ckd") OR (AB "ckd") |
| S27 | (TI "ckf") OR (AB "ckf") |
| S28 | (TI "crd") OR (AB "crd") |
| S29 | (TI "crf") OR (AB "crf") |
| S30 | (TI "eskd") OR (AB "eskd") |
| S31 | (TI "eskf") OR (AB "eskf") |
| S32 | (TI "esrd") OR (AB "esrd") |
| S33 | (TI "esrf") OR (AB "esrf") |
| S34 | S9 OR S10 OR S11 OR S12 OR S13 OR S14 OR S15 OR S16 OR S17 OR S18 OR S19 OR S20 OR S21 OR S22 OR S23 OR S24 OR S25 OR S26 OR S27 OR S28 OR S29 OR S30 OR S31 OR S32 OR S33 |
| S35 | S8 AND S34 |
|  |  |

**Cochrane Central Register of Controlled Trials (CENTRAL) Search Strategy**

**Platform**: Wiley

**Years Searched**: Issue 4 of 12, April 2021; Issue 2 of 12, February 2022

**Limits**: None

**Dates Searched:** May 17, 2021; March 4, 2022

**Number of Results:** 39; 42

| 1 | [mh clostridioides] |
| --- | --- |
| 2 | [mh ^clostridium] |
| 3 | [mh "clostridium infections"] |
| 4 | ((bacillus OR clostrid* OR peptoclostrid* OR pseudomembran*) NEAR/2 (colitis OR difficile OR dificile OR difficilis OR dificilis OR diarrhea OR diarrhoea OR disease* OR enteritis OR enterocolitis OR infect*)):ti,ab,kw |
| 5 | (clostridioses):ti,ab,kw |
| 6 | (clostridiosis):ti,ab,kw |
| 7 | ("c diff"):ti,ab,kw |
| 8 | (cdad):ti,ab,kw |
| 9 | {OR #1-#8} |
| 10 | [mh "renal replacement therapy"] |
| 11 | [mh ultrafiltration] |
| 12 | ("renal replacement" NEAR/2 (continuous OR therap*)):ti,ab,kw |
| 13 | (dialys*):ti,ab,kw |
| 14 | (h?emodiafiltrat*):ti,ab,kw |
| 15 | (h?emodialys*):ti,ab,kw |
| 16 | (h?emofiltrat*):ti,ab,kw |
| 17 | ("acetate free" NEAR/2 biofiltrat*):ti,ab,kw |
| 18 | (ultrafiltrat*):ti,ab,kw |
| 19 | (cavhd):ti,ab,kw |
| 20 | (crrt):ti,ab,kw |
| 21 | (cvvh):ti,ab,kw |
| 22 | (cvvhd):ti,ab,kw |
| 23 | (cvvhdf):ti,ab,kw |
| 24 | (scuf):ti,ab,kw |
| 25 | [mh "renal insufficiency, chronic"] |
| 26 | ((kidney OR renal) NEAR/2 (chronic OR "end stage" OR endstage)):ti,ab,kw |
| 27 | (ckd):ti,ab,kw |
| 28 | (ckf):ti,ab,kw |
| 29 | (crd):ti,ab,kw |
| 30 | (crf):ti,ab,kw |
| 31 | (eskd):ti,ab,kw |
| 32 | (eskf):ti,ab,kw |
| 33 | (esrd):ti,ab,kw |
| 34 | (esrf):ti,ab,kw |
| 35 | {OR #10-#34} |
| 36 | {AND #9, #35} |
