# Supplementary material for: Prevalence and trends of Clostridioides difficile infection among persons requiring maintenance hemodialysis: A systematic review and meta-analysis
Source: Infect Control Hosp Epidemiol. 2022 Sep 23;44(7):1068–75. doi: 10.1017/ice.2022.217 (PMC10369223; doi:10.1017/ice.2022.217)
Supplement: Supplementary file 1 [file S0899823X22002173sup.zip › S0899823X22002173sup002.docx]

**Supplementary Table 1.** Newcastle-Ottawa scale for assessment of quality of included studies – case control studies (each asterisk represents if individual criterion within the subsection was fulfilled). N/A: not applicable; MHD: maintenance hemodialysis

| **Quality assessment criteria** | **Acceptable (★)** | **D’Agata**  **2020** | **Eui Oh**  **2013** | **Huang**  **2014** | **Morfin**  **2018** | **Predrag**  **2016** | **Wei**  **2015** |
| --- | --- | --- | --- | --- | --- | --- | --- |
| Is the case definition adequate? | Yes, with independent validation | ★ | ★ | ★ | ★ | ★ | ★ |
| Representative-ness of cases | Consecutive or obviously representative series of cases | ★ | ★ | ★ | ★ | ★ | ★ |
| Selection of controls | Hospital controls | ★ | ★ | ★ | ★ | ★ | ★ |
| Definition of controls | Not requiring MHD | ★ | ★ | ★ | ★ | ★ | No |
| Study controls for patient factors | N/A | N/A | N/A | N/A | N/A | N/A | N/A |
| Ascertainment of exposure? | Yes | ★ | ★ | ★ | ★ | ★ | ★ |
| Same method of ascertainment of cases/controls? | Yes | ★ | ★ | ★ | ★ | ★ | ★ |
| Non-response rate | Same rate for both groups | ★ | ★ | ★ | ★ | ★ | ★ |
| Overall Quality score (maximum = 7) | | 7 | 7 | 7 | 7 | 7 | 6 |
