# Supplementary material for: Prevalence and trends of Clostridioides difficile infection among persons requiring maintenance hemodialysis: A systematic review and meta-analysis
Source: Infect Control Hosp Epidemiol. 2022 Sep 23;44(7):1068–75. doi: 10.1017/ice.2022.217 (PMC10369223; doi:10.1017/ice.2022.217)
Supplement: Supplementary file 1 [file S0899823X22002173sup.zip › S0899823X22002173sup003.docx]

**Supplementary Table 2.** Newcastle-Ottawa scale for assessment of quality of included studies – cohort studies (each asterisk represents if individual criterion within the subsection was fulfilled). N/A: not applicable

| **Quality assessment criteria** | **Acceptable (★)** | **Demir**  **2018** | **Keddis**  **2012** |
| --- | --- | --- | --- |
| Selection | | | |
| Representative of the exposed cohort | Truly representative of the average in the community | ★ | ★ |
| Selection of the non- exposed cohort |  | ★ | ★ |
| Ascertainment of exposure | Secure record or structured interview | ★ | ★ |
| Demonstration that outcome of interest was not present at start of study | Yes | ★ | ★ |
| Comparability | | | |
| Study controls for patient factors | N/A | N/A | N/A |
|  | Study control for any additional factor | No | ★ |
| Outcome | | | |
| Assessment of outcome | Independent blind assessment or record linkage | ★ | ★ |
| Was follow-up long enough for outcome to occur | Yes | ★ | ★ |
| Adequacy of follow up of cohorts | Complete follow up or subjects lost to follow up unlikely to introduce bias | ★ | ★ |
| Overall Quality score (maximum = 7) | | 6 | 7 |
