# Supplementary figures and images for: Prevalence and trends of Clostridioides difficile infection among persons requiring maintenance hemodialysis: A systematic review and meta-analysis
Source: Infect Control Hosp Epidemiol. 2022 Sep 23;44(7):1068–75. doi: 10.1017/ice.2022.217 (PMC10369223; doi:10.1017/ice.2022.217)

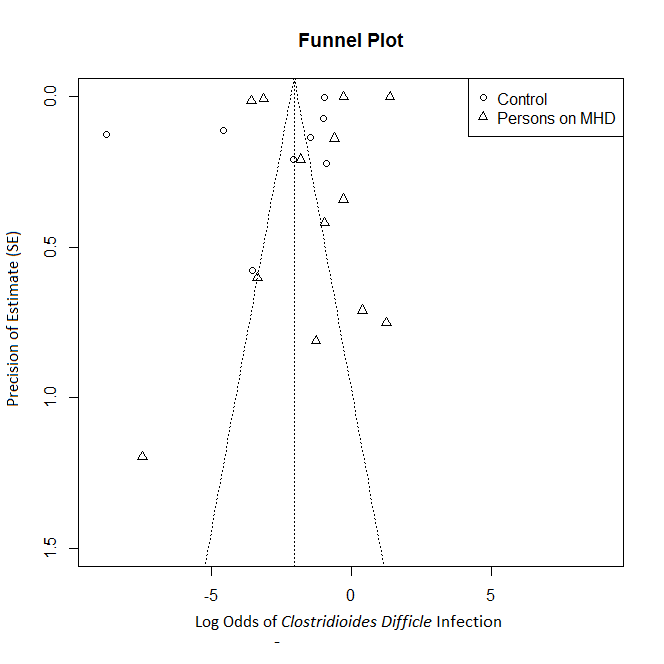

Supplement: Supplementary file 1 [file S0899823X22002173sup.zip › S0899823X22002173sup004.tif]
